# The protective effects of memantine against inflammation and impairment of endothelial tube formation induced by oxygen-glucose deprivation/reperfusion

**DOI:** 10.18632/aging.103914

**Published:** 2020-11-07

**Authors:** Xiaoxin Lv, Qiang Li, Shuai Mao, Limin Qin, Peikang Dong

**Affiliations:** 1 Department of Cardiology, Affiliated Hospital of Weifang Medical University, Weifang 261031, Shandong, China

**Keywords:** myocardial infarction, OGD/R, memantine, NMDA-receptor antagonist I, microtubule formation

## Abstract

Acute myocardial infarction (AMI) is one of the leading causes of death and disability. The dysregulation of cardiac endothelial cells plays a significant role in the pathogenesis of AMI. In the present study, we investigated the potential of memantine, a noncompetitive N-methyl-D-aspartate (NMDA) receptor antagonist used in the treatment of Alzheimer’s disease, to mitigate the effects of ischemia-reperfusion injury in the peripheral vasculature using human umbilical cord endothelial cells (HUVECs). Previous studies have identified anti-inflammatory and antioxidant effects of memantine, but the effects of memantine on angiogenesis and microtubule formation have not been fully elucidated. Our findings indicate that pretreatment with memantine significantly reduced the expression of interleukin (IL)-6 and IL-8, which are both serum markers if AMI severity. We also demonstrate that memantine could prevent mitochondrial dysfunction and oxidative stress by rescuing mitochondrial membrane potential and reducing the production of reactive oxygen species (ROS) by NADPH oxidase-4 (NOX-4). Importantly, memantine also promoted the expression of the nuclear factor erythroid 2-related factor 2 (Nrf2)/heme oxygenase-1 (HO-1) antioxidant signaling pathway. Importantly, memantine pretreatment improved cell viability and prevented the decrease in microtubule formation induced by OGD/R. Through a phosphoinositide-3-kinase (PI3K) inhibition experiment, we determined that the PI3K/protein kinase B (Akt) pathway is essential for the effects of memantine on angiogenesis. Together, our findings suggest a potential role for memantine in the prevention and treatment of AMI.

## INTRODUCTION

Globally, acute myocardial infarction (AMI) is one of the leading causes of death and the number one cause of disability. It is projected that the financial and public health burden of AMI will nearly double over the next ten years [1]. In AMI, a blood vessel most often becomes partially or totally occluded, resulting in potentially severe deprivation of vital oxygen and glucose to the tissue of the heart, thereby inducing acute vascular and endothelial cell death and irreversible tissue damage [2]. Common causes of AMI include atherosclerosis, interarterial embolization, and blood clots, among other things [3]. To date, there are few options for the treatment of this disease due to its complex pathophysiology.

Immediately following the occurrence of AMI, areas starved of oxygen and nutrients suffer acute myocardiocyte cell death, which triggers an intercellular signaling cascade. This results in an acute inflammatory response, degradation of the extracellular matrix (ECM), scar tissue formation, ventricular dilation, and progressive tissue remodeling. These events can eventually lead to heart failure [4]. Meanwhile, endothelial cells are activated in response to ischemic injury to trigger angiogenesis, thereby aiding in the restoration of cardiac function [5]. Another critical event that occurs immediately following AMI is an acute inflammatory reaction in response to circulatory stagnation. Proinflammatory factors, such as interleukin (IL)-6, IL-8, tumor necrosis factor (TNF)-α, and others, are released by endothelial cells upon activation by harmful stimuli such as hypoxia [6–7]. This inflammatory response aids in the recovery of cardiac function, but an excessive or extended inflammatory response leads to further myocardial injury [8]. Increased serum levels of IL-6 have long been associated with poorer outcomes in AMI patients [9]. Similarly, high serum levels of IL-8 are associated with more severe disability after acute AMI [10]. Thus, therapies that aim to mitigate the harmful effects of acute inflammation while promoting their reparatory role are of considerable value.

Oxidative stress and mitochondrial damage are also defining aspects of AMI injury. Reoxygenation following hypoxia induces an abrupt increase in the production of reactive oxygen species (ROS), which is mediated by NADPH oxidase-4 (NOX-4) and exceeds the capacity of the body’s antioxidant system. Reducing the expression of NOX-4 and the generation of ROS is considered a promising therapeutic approach for AMI [11]. Nuclear factor erythroid 2-related factor 2 (Nrf2) is a ubiquitously expressed transcription factor containing 605 amino acids. Nrf2 regulates free radical scavenging and redox homeostasis while heme oxygenase-1 (HO-1), its downstream target, maintains cellular redox functionality. Nrf2 and HO-1 have been shown to confer protective effects against AMI in mouse models by preventing excessive ROS production, promoting the function of the antioxidant system, and reducing markers of heart failure [12–14]. Phosphoinositide-3-kinase (PI3K) and its downstream kinase protein kinase B (Akt) have been shown to confer protective effects against AMI by reducing oxidative stress and inflammation, and regulating myocardial cell survival and autophagic flux [15–16]. Angiogenesis following AMI is known to improve patient outcome. Recent research has suggested promoting the activation of Nrf2 and the PI3K/Akt pathway as a treatment for AMI [17].

Memantine is a noncompetitive N-methyl-D-aspartate (NMDA) receptor antagonist licensed by the FDA for the treatment of Alzheimer’s disease that has recently been gaining attention for its potential in the treatment of AMI [18]. For example, memantine has been shown to attenuate cardiac remodeling by reducing myocyte necrosis, improving the heart weight to body weight ratio, and inhibiting lipid peroxidation [19]. Additionally, a contemporary study found that there was a significantly lower rate of hospital admissions for heart failure among patients taking memantine, suggesting a protective role [20]. However, the mechanisms involved in memantine-mediated cardiac protection are complicated and remain incompletely understood. In the present study, we investigated the effects of memantine in human umbilical vein endothelial cells (HUVECs) exposed to oxygen-glucose deprivation and reoxygenation (OGD/R) to simulate AMI. We measured the expression of inflammatory cytokines, oxidative stress, and Nrf2/HO-1 signaling. We also assessed the involvement of the PI3K/Akt pathway in mediating memantine-induced angiogenesis. Our findings provide a greater understanding of the protective effects of memantine against AMI.

## RESULTS

### Molecular structure of memantine

**Figure 1:**
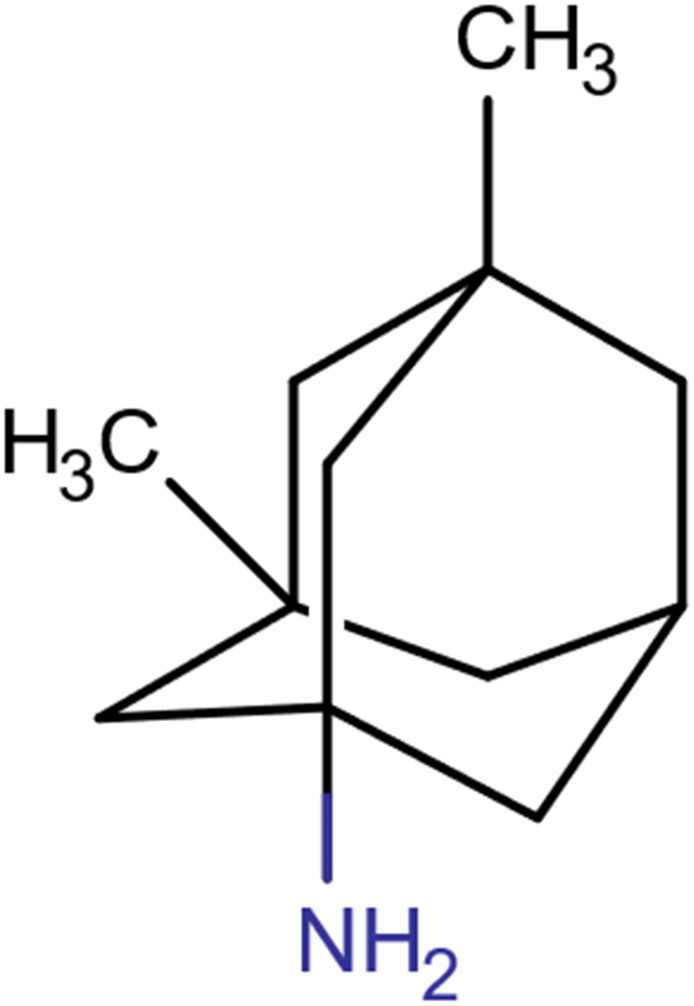
**Molecular structure of memantine.**

Memantine (3,5-dimethyladamantan-1-amine hydrochloride) is a low-affinity, voltage-dependent, noncompetitive NMDA ion channel blocker with the molecular formula C_12_H_22_ClN and a molecular weight of 215.76 g/mol. The molecular structure of memantine is shown in Figure 1. Memantine is symmetrical in shape and has been shown to bind to NMDA receptors by blocking the ion channel via interaction with asparagine residues found on the receptor pore loops [21].

### Anti-inflammatory effects of memantine

**Figure 2:**
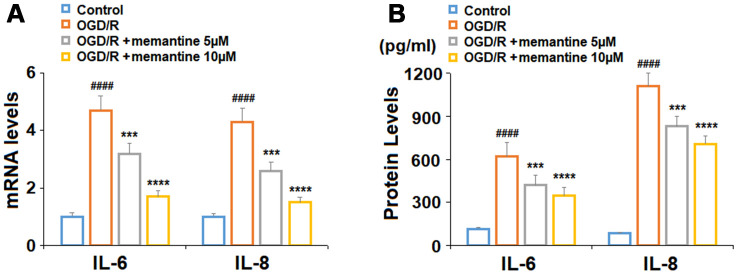
**Memantine suppressed oxygen-glucose deprivation/reperfusion-induced expression and secretions of proinflammatory cytokines in human umbilical vein endothelial cells (HUVECs).** Cells were treated with memantine (5, 10 μM) for 6 h, followed by exposure to oxygen-glucose deprivation (6 h)/reperfusion (24 h) (OGD/R). (**A**) mRNA levels of IL-6 and IL-8; (**B**). Secretions of IL-6 and IL-8 (####, P<0.0001 vs. vehicle group; ***, *****, P<0.001, 0.0001 vs OGD/R group).

For our first experiment, we determined whether memantine could reduce the expression of the two key inflammatory cytokines : IL-6 and IL-8. While exposure to OGD/R induced an increase in IL-6 and IL-8 mRNA expression to 4.7- and 4.3-fold, respectively, 5 and 10 μM memantine dose-responsively reduced their expression to 1.7- and 1.5-fold (Figure 2A). At the protein level, the two doses of memantine also inhibited the increase in IL-6 and IL-8 induced by OGD/R (Figure 2B). Thus, memantine significantly reduces the expression of these two key inflammatory cytokines induced by OGD/R at both the mRNA and protein levels.

### Memantine restores mitochondrial membrane potential

**Figure 3:**
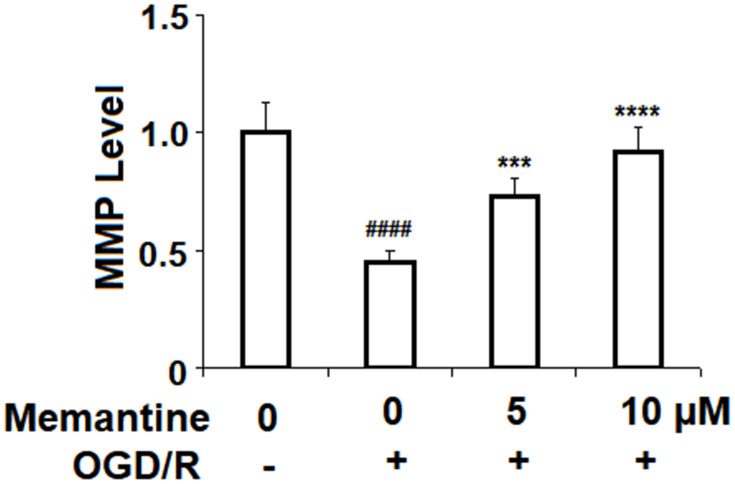
**Memantine restored oxygen-glucose deprivation/reperfusion-induced reduction of mitochondrial membrane potential (ΔΨm) in human umbilical vein endothelial cells (HUVECs).** Cells were treated with memantine (5, 10 μM) for 6 h, followed by exposure to oxygen-glucose deprivation (6 h)/reperfusion (24 h) (OGD/R). Intracellular ΔΨm was measured by rhodamine 123 (RH123) (####, P<0.0001 vs. vehicle group; ***, *****, P<0.001, 0.0001 vs OGD/R group).

Next, we assessed the effects of memantine on OGD/R-induced reduced mitochondrial membrane potential (ΔΨm). Mitochondrial membrane potential is known to play a central role in the generation of ROS and the regulation of cell survival and apoptosis. In Figure 3, we demonstrate that while OGD/R reduced ΔΨm by more than half, memantine dose-responsibly restored ΔΨm to 92%.

### Memantine reduces ROS production by NOX-4

**Figure 4:**
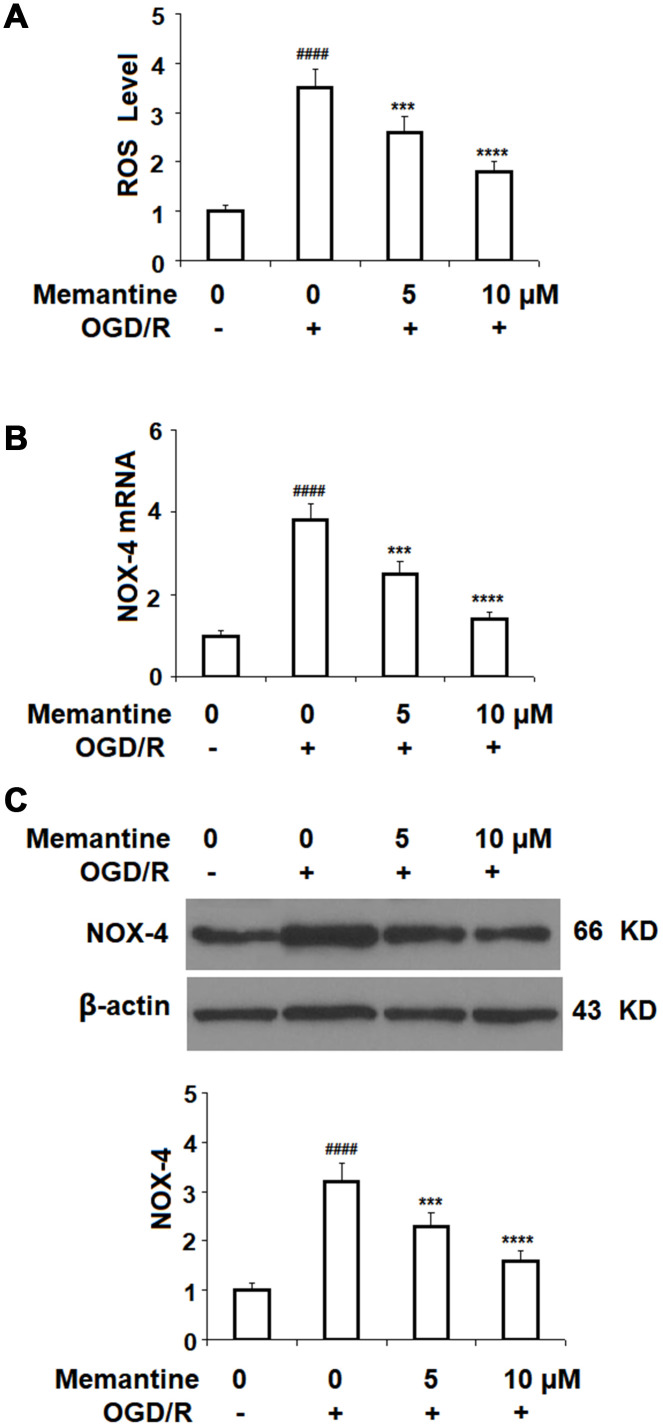
**Memantine suppressed oxygen-glucose deprivation/reperfusion-induced oxidative stress in human umbilical vein endothelial cells (HUVECs).** Cells were treated with memantine (5, 10 μM) for 6 h, followed by exposure to oxygen-glucose deprivation (6 h)/reperfusion (24 h) (OGD/R). (**A**) Intracellular ROS was measured by dihydroethidium (DHE); (**B**) mRNA of NOX-4 as measured by real-time PCR; (**C**) Protein of NOX-4 as measured by western blot analysis (####, P<0.0001 vs. vehicle group; ***, *****, P<0.001, 0.0001 vs OGD/R group).

Next, we measured the effect of memantine of OGD/R-induced ROS generation. The results in Figure 4A show that while OGD/R increased the level of intracellular ROS 3.5-fold, the two doses of memantine reduced the level of ROS to 2.6- and 1.8-fold, respectively. NOX-4 is an important producer of ROS. We employed real-time PCR and western blot analyses to determine whether the reduction in ROS is mediated through suppression of NOX-4. Indeed, the results in Figure 4A and 4B indicate that the increase in NOX-4 expression was inhibited by memantine at both the mRNA and protein levels in a dose-responsive manner. Thus, memantine significantly reduces the oxidative stress response in an *in vitro* model of IS.

### Memantine increases the expression of Nrf2 and HO-1

**Figure 5:**
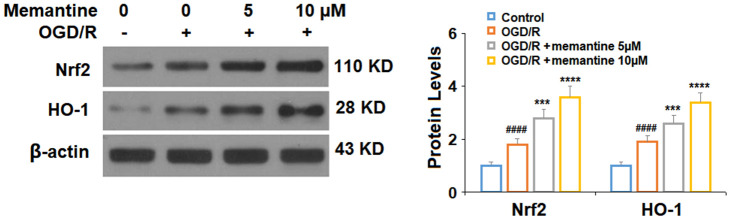
**Memantine increased the expression of Nrf2 and HO-1 under OGD/R in HUVECs.** Cells were treated with memantine (5, 10 μM) for 6 h, followed by exposed to oxygen-glucose deprivation (6 h)/reperfusion (24 h) (OGD/R). Expressions of Nrf2 and HO-1 were measured by western blot analysis (####, P<0.0001 vs. vehicle group; ***, *****, P<0.001, 0.0001 vs OGD/R group).

Next, we measured the effects of memantine of the expression Nrf2 and HO-1, two protective factors involved in AMI. In agreement with previous research using HUVECs [22], we found that exposure to OGD/R increased the protein expression levels of both Nrf2 and HO-1 by around 85%. Here, we show that the 5 and 10 μM memantine further increased the expression of these two factors to roughly 350% in a dose-responsive manner (Figure 5). These findings suggest that the significant protective effects of memantine against oxidative stress may be mediated through the activation of the Nrf2/HO-1 redox system.

### Memantine improves cell viability

**Figure 6:**
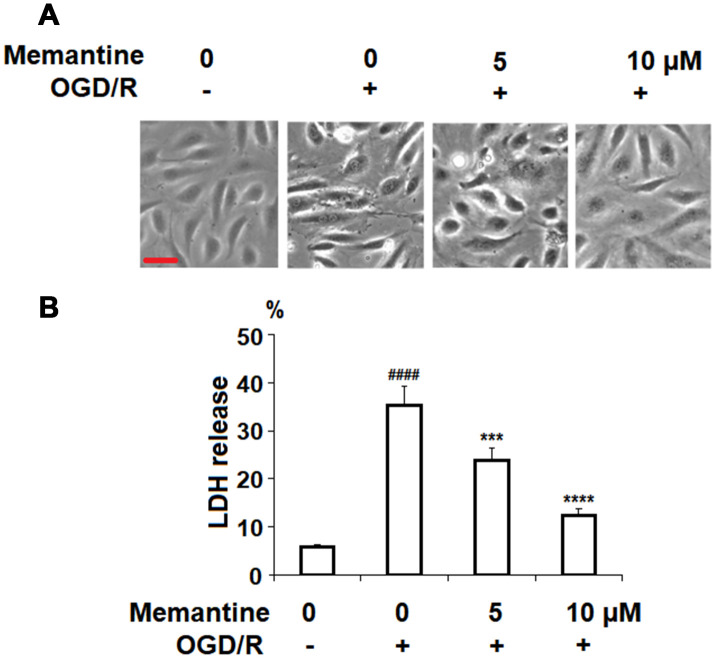
**Memantine protected oxygen-glucose deprivation/reperfusion (OGD/R)-induced cell death in HUVECs.** Cells were treated with memantine (5, 10 μM) for 6 h, followed by exposure to oxygen-glucose deprivation (6 h)/reperfusion (24 h) (OGD/R). (**A**) Cell morphology of HUVECs; Scale bar, 100 μm; (**B**) LDH release (####, P<0.0001 vs. vehicle group; ***, *****, P<0.001, 0.0001 vs OGD/R group).

Next, we investigated the effects of memantine on OGD/R-induced cell death by measuring the release of LDH. LDH is a cytoplasmic enzyme released from damaged cells and detecting the release of LDH is a widely used method for measuring the rate of cell death [22]. The cell morphology of HUVECs in the different groups is shown in Figure 6A. Here, we found that while OGD/R significantly increased the release of LDH to roughly 5.5-fold, the two doses of memantine ameliorated this effect, as evidenced by the dose-dependent reduction to 4-fold and 2-fold, respectively (Figure 6B).

### Memantine promotes angiogenesis in HUVECs

**Figure 7:**
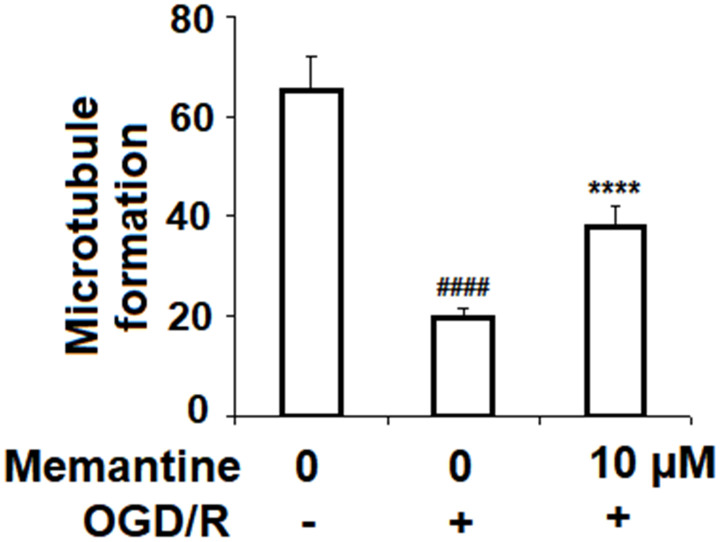
**Memantine prevented OGD/R-induced reduced angiogenesis in HUVECs.** Cells were treated with memantine (10 μM) for 6 h, followed by exposure to oxygen-glucose deprivation (6 h)/reperfusion (24 h) (OGD/R). Microtubule formation was quantified by Matrigel assay (####, P<0.0001 vs. vehicle group; *****, P<0.0001 vs OGD/R group).

To determine the effects of memantine on angiogenesis, HUVECs were subjected to the OGD/R in the presence or absence of 10 μM memantine, and then a Matrigel assay was used to quantify the formation of microtubules. As shown in Figure 7, microtubule formation was reduced by 70%, while this reduction was only 42% when the cells were treated with memantine. Thus, memantine might protect against OGD/R-induced reduced angiogenesis.

**Figure 8:**
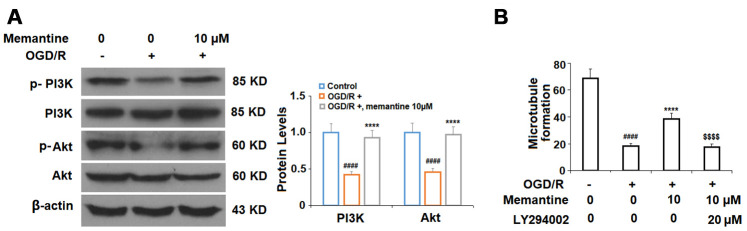
**The effects of memantine on endothelial microtubule formation are mediated by the PI3K/Akt signaling in HUVECs.** (**A**) Cells were treated with memantine (10 μM) for 6 h, followed by exposure to oxygen-glucose deprivation (6 h)/reperfusion (24 h) (OGD/R). Phosphorylated and total levels of PI3K and Akt were measured; (**B**) Inhibition of PI3K/Akt with LY294002 (20 μM) abolished the protective effects of memantine in microtubule formation (####, P<0.0001 vs. vehicle group; *****, P<0.0001 vs OGD/R group; $$$$, P<0.0001 vs. OGD/R+ memantine group).

**Figure 9:**
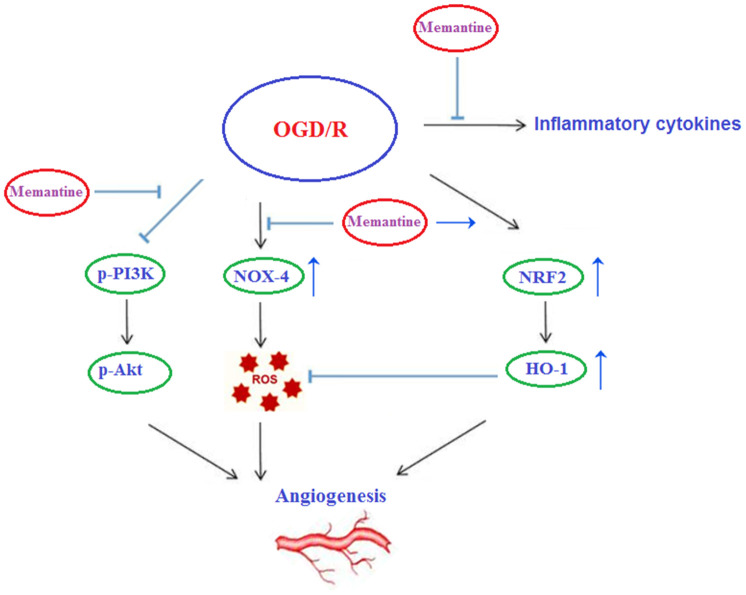
**Graphic summary of the underlying mechanism.**

Finally, we determined whether the effects of memantine on microtubule formation were mediated by the PI3K/Akt pathway. Here, we employed the PI3K inhibitor LY294002. PI3K constitutes an enzyme family that phosphorylates the 3′-OH of the phosphatidylinositol inositol ring, while Akt regulates protein synthesis and cell growth via phosphorylation of mammalian target of rapamycin (mTOR). The PI3K/Akt pathway promotes angiogenesis by increasing the expression of vascular endothelial growth factor (VEGF) and modulating the expression of nitric oxide and angiopoietins, which are essential for angiogenesis [23]. Here, we found that 10 μM memantine rescued the levels of phosphorylated PI3K and Akt to near baseline as compared to a reduction of roughly 55% induced by OGD/R (Figure 8A). Furthermore, we found that inhibition of PI3K by 20 μM LY294002 abolished the effects of memantine on microtubule formation (Figure 8B). Thus, memantine promotes angiogenesis during OGD/R by activating the PI3K/Akt pathway. A graphic summary of the underlying mechanism is shown in Figure 9.

## DISCUSSION

The present study investigated the potential protective role of the NMDA receptor antagonist memantine in the peripheral vasculature using an *in vitro* HUVEC model of AMI. There has been increasing evidence regarding the protective effects of memantine against ischemic injury. For example, a contemporary study found that antagonism of NMDA receptors by memantine could protect against reperfusion injury and disruption of the blood-brain barrier (BBB) following acute ischemic stroke in mice with mild hyperhomocysteinemia [24]. Memantine has also been shown to reduce the expression of matrix metalloproteinases and increase the expression of the well-known protective transcriptional pathway Kruppel-like factor 2 (KLF2) [25]. Another study showed that memantine could prevent brain infarct and neuronal injury while reducing neuronal apoptosis induced by cerebral ischemia [26]. However, the effects of memantine on cardiac tissue exposed to hypoxia/reoxygenation have not been thoroughly studied. Importantly, promoting angiogenesis and neovascularization following AMI is considered an effective therapy for improving patient recovery. The return of blood flow to the ischemic area has been shown to prevent cardiomyocyte apoptosis, fibrosis, and heart failure by promoting long-term remodeling of the left ventricle [27]. In our experiments, we investigated the capacity of memantine to protect against OGD/R-induced damage in HUVECs, a cell type that been widely used in similar studies [28]. Our findings show that memantine may indeed confer protective effects against ischemic injury in the peripheral vasculature.

Memantine has demonstrated anti-inflammatory effects in several tissues and cell types. For example, a study using a mouse model of chronic obstructive pulmonary disease found that memantine could inhibit the expression of IL-6, TNF-α, and interferon-γ, thereby reducing pulmonary inflammation [29]. Another study showed an anti-inflammatory capacity of memantine in a rat model of peptic ulcer [30]. Here, we demonstrated that pretreatment with memantine could significantly inhibit the expression of IL-6 and IL-8, two key indicators of AMI severity and disability, at both the mRNA and protein levels. We also found that memantine pretreatment could attenuate oxidative stress induced by OGD/R. Congruent with the results of our research, numerous studies have highlighted the antioxidant functions of memantine [31, 32]. NMDA has been shown to induce oxytosis, a form of nonapoptotic cell death characterized by oxidative stress and mitochondrial dysfunction [33]. Additionally, NMDA antagonism has been shown to ameliorate some of the deleterious effects of ischemia/reperfusion injury in an *ex vivo* rat heart model of AMI [34]. Notably, we demonstrate that memantine rescued the reduction in mitochondrial membrane potential and inhibited oxidative stress in HUVECs induced by OGD/R. Specifically, memantine reduced the production of ROS, inhibited NOX-4 expression, and enhanced the activity of the Nrf2/HO-1 antioxidant pathway. Increasing the expression of HO-1 through Nrf2 is considered a valuable therapeutic approach, as Nrf2 activation rapidly increases HO-1 expression, thereby protecting cells from oxidative, excitotoxic, and inflammatory damage [35]. Other studies have demonstrated the importance of Nrf2/HO-1 signaling in AMI both *in vitro* and *in vivo* [36, 37]. However, the research regarding the effects of memantine on Nrf2/HO-1 activation remains rather limited. Our findings provide a basis for further exploration of memantine-mediated Nrf2/HO-1 activation.

Angiogenesis and neovascularization are key events in AMI recovery. In AMI, increasing the activation of the PI3K/Akt pathway is well-recognized as a therapeutic approach to promoting angiogenesis. Interestingly, knockdown of Nrf2 results in reduced angiogenesis via inhibition of the PI3K/Akt pathway [38]. Additionally, NMDA receptor antagonism has been shown to modulate the expression of the PI3K/Akt pathway [39]. Here, we found that memantine pretreatment remarkably protected against OGD/R-induced reduced microtubule formation and that this effect was dependent on PI3K signaling. Indeed, a contemporary study demonstrates that pretreatment with the novel memantine derivative memantine nitrate-06 (MN-06) can rescue the function of the PI3K/Akt pathway [40]. Meanwhile, earlier research demonstrated an ability of memantine to prevent inhibition of the PI3K/Akt/mTOR pathway in an APP695-overexpressing SH-SY5Y cell model of Alzheimer’s disease [41]. These findings support our theory that memantine might serve as a valuable treatment to promote angiogenesis in peripheral tissues following AMI.

There are several limitations to the current study. Namely, an *in vitro* cell model of AMI was used to evaluate the effects of memantine. While this approach allowed us to observe specific mechanisms in a controlled environment, it is unclear how our findings will translate to whole organisms. Thus, we have ongoing studies in our lab involving animal models, which will be reported on in our next full paper. Additionally, there are numerous other mechanisms and signaling pathways involved in angiogenesis, including cytokines, integrins, growth factors, and microRNAs, among other things [42]. Here, we only investigated a select few. Our findings lay the groundwork for further research in this area. Finally, while OGD/R is a widely used model of AMI [43], the processes of AMI and ischemia-reperfusion injury are complicated and may not all be taken into account. This will also be addressed in our ongoing animal studies.

Taken together, our findings demonstrate the potential of pretreatment with memantine, a noncompetitive NMDA receptor inhibitor, to protect against damages associated with AMI while promoting angiogenesis. Memantine suppressed inflammation, as evidenced by reduced expression of IL-6 and IL-8, two important serum markers of AMI severity. Memantine not only rescued the reduction in mitochondrial membrane potential induced by OGD/R, but also decreased oxidative stress by inhibiting the production of ROS by NOX-4 and increasing the activity of the Nrf2/HO-1 antioxidant pathway. The results of our cell viability assay demonstrate a good safety profile of memantine in HUVECs. Finally, memantine prevented the reduction in microtubule formation induced by OGD/R, which was dependent on the PI3K/Akt pathway. Thus, memantine may confer valuable protective effects against AMI while promoting recovery through increased angiogenesis.

## MATERIALS AND METHODS

### Cell culture and treatment

Experiments were designed in accordance with the World Medical Association Declaration of Helsinki Ethical Principles for Medical Research Involving Human Subjects. Experimental procedures were approved by the institutional ethics committee at Weifang Medical University (WFU-E20170039). Human umbilical vein endothelial cells (HUVECs) purchased from the American Type Culture Collection (ATCC) were cultured in complete EGM-2 media containing 2% FBS, and maintained in a 5% CO_2_ humidified incubator at 37 °C. The cells were harvested at passages 3–7. For pretreatment, 5 and 10 μM memantine (Cat#1380502, Sigma-Aldrich, USA) [25] were added to the culture media for 6 hours. Once the cells had reached 95% confluence, they were washed 3 times with PBS and the culture medium was replaced with glucose-free EGM. The cells were then transferred to an anaerobic atmosphere (95% N_2_/5% CO_2_) and incubated at 37 °C for 6 hours. For reoxygenation, HUVECs were moved to glucose-containing media and incubated for an additional 24 hours under normal culture conditions. For our PI3K/Akt inhibition experiment, the cells were pretreated with 20 μM LY294002 (Cat#HY-D0816, MedchemExpress) [44] for 6 hours.

### Real-time PCR

Total RNA was extracted from HUVECs using TRIzol (Invitrogen Life Technologies, USA) in accordance with the manufacturer’s instructions. First-strand cDNA was synthesized using a PrimeScript RT Kit (Takara Biotechnology Co., Ltd., Dalian, China). A TaqMan Assay Kit (Thermo Fisher Scientific, Inc.) was used to perform RT-PCR. Then, the mRNA levels of IL-6, IL-8, and NOX-4 were detected using SYBR Green real-time PCR method. The amplification conditions for PCR were: denaturation at 95 °C for 10 s; 40 cycles at 95 °C for 5 s; 40 cycles at 60 °C for 20 s. The results are expressed as fold changes and were determined using the 2^−ΔΔCT^ method [45]. The following primers were used in this study: IL-6: (Forward, 5’-AGGGCTCTTCGGGAAATGTA-3’, Reverse, 5’CTTGACGGTGCCATGGAATT3’); IL-8: (Forward, 5’-TTTCTGTTAAATCTGGCAACCCTAGT-3’, Reverse, 5’- ATAAAGGAGAAACCAAGGCACAGT-3’); NOX-4: (Forward, 5’-CAGATGTTGGGGCTAGGATTG-3’, Reverse, 5’- GAGTGTTCGGCACATGGGTA-3’). GAPDH: (Forward, 5’-ACTGGCGTCTTCACCACCAT-3’, Reverse, 5’-AAGGCCATG CCAGTGAGCTT-3’).

### Western blot analysis

HUVECs were lysed in ice-cold RIPA buffer (20 mM Tris–HCl (pH 7.5), 150 mM NaCl, 1 mM Na2EDTA, 1 mM EGTA, 1% NP-40, 1% sodium deoxycholate) supplemented with proteinase and phosphatase inhibitors for 30 min to obtain cell lysates. Debris was removed by centrifuging the lysates at 14,000 × rpm at 4 °C, and a BCA Protein Assay Kit (Sigma-Aldrich, USA) was used to quantify the protein concentration. Next, equal amounts of protein were separated onto a 10% SDS-PAGE gel and then transferred onto PVDF membranes. The membranes were blocked with 5% non-fat milk and incubated with primary antibodies overnight at 4 °C. The following day, the membranes were incubated with horseradish peroxidase-conjugated secondary antibodies. Enhanced chemiluminescence reagents (ECL) (Cat#PI80196, Thermo Fisher Scientific, USA) was used to develop the blots, which were read using a Canon scanner. Image J software (NIH) was used to normalize the density the bands to β-actin [46].

### ELISA

To determine the protein concentrations of IL-6 (Cat#D6050, R&D Systems) and IL-8 (Cat#D8000C, R&D Systems), ELISA kits were used in accordance with the manufacturer’s instructions. Briefly, the cells underwent the indicated treatment, and then the culture media was collected for analysis. The data were collected using a 96-well plate reader spectrometry, and a standardized 4-PL curve was used to obtain absolute values. The relative levels of the target genes are presented as normalized to total protein amounts.

### Determination of mitochondrial membrane potential

Intracellular ΔΨm was measured using the cationic dye rhodamine 123 (RH123) as previously described [47]. Briefly, RH123 (Cat#R8004, Sigma-Aldrich, USA) was dissolved in alcohol and then used to stain the cells. Then, a laser-scanning confocal microscope (Leica TCS SP2) was used to visualize the stained cells. The fluorescence intensity of the dye was quantitated using Image J software (NIH, USA). The levels of intracellular ΔΨm were calculated using the software Image J. Firstly, we defined regions of interest (ROIs) and counted the average number of cells (N) in the ROIs. Secondly, the integrated density value (IDV) in ROIs was then calculated. Average levels of intracellular ΔΨm =IDV/N.

### Determination of ROS

The cell-permanent dye dihydroethidium (DHE) (Cat#D7008, Sigma-Aldrich, USA) was used to determine the production of ROS in HUVECs. After OGD/R with or without memantine pretreatment, the cells were washed 3 times with PBS and then stained with DHE. The cells were incubated with 10 μM DHE at 37 °C for 30 minutes in darkness. Then, a laser-scanning confocal microscope (Leica TCS SP2) was used to visualize the stained cells. The fluorescence intensity of the dye was quantitated using Image J software (NIH, USA). The levels of intracellular ROS were calculated using the software Image J. Firstly, we defined regions of interest (ROIs) and counted the average number of cells (N) in the ROIs. Secondly, the integrated density value (IDV) in ROIs was then calculated. Average levels of intracellular ROS=IDV/N.

### Measurement of LDH release

Cell cytotoxicity was determined based on the release of lactate dehydrogenase (LDH). Briefly, 1 × 10^4^ HUVECs were seeded onto a 96-well plate and then exposed to the necessary treatment. The cell culture media was collected and centrifuged to obtain supernatant for analysis. The rate of LDH release was detected using a commercial LDH Assay Kit (EPX010-12262-901, Thermo Fisher Scientific, USA) in accordance with the manufacturer’s instructions. The reaction was read at 490 nm. The rate of LDH release was normalized to the non-treated cells.

### Matrigel assay

To assess endothelial tube formation, HUVECs were cultured on a commercial Matrigel matrix (BD Biosciences). Briefly, 5000 cells were plated onto a 24-well plate precoated with 6 mg mL−1 Matrigel membrane at 4 °C. The cells were incubated for 72 hours at 37 °C. Ten random fields of interest were selected for imaging. The resulting images were analyzed, and tube formation was quantified using Image J software (NIH, USA).

### Statistics

Prism version 5 (GraphPad Software) was used to analyze the results of our experiments. All experimental data are presented as means ± S.E.M. Comparisons between more than two groups were made using one-way ANOVA followed by Dunnett’s multiple comparisons post-hoc test. A P-value of less than 0.05 was considered to represent statistical significance.
